# Do Cervical Cancer Patients Diagnosed with Opportunistic Screening Live Longer? An Arkhangelsk Cancer Registry Study

**DOI:** 10.3390/ijerph14121500

**Published:** 2017-11-26

**Authors:** Elena E. Roik, Evert Nieboer, Olga A. Kharkova, Andrej M. Grjibovski, Vitaly A. Postoev, Jon Ø. Odland

**Affiliations:** 1 Department of Community Medicine, Faculty of Health Sciences, UiT—The Arctic University of Norway, Tromsø N-9037, Norway; olga.a.kharkova@uit.no (O.A.K.); jon.oyvind.odland@uit.no (J.Ø.O.); 2 International School of Public Health, Northern State Medical University, Arkhangelsk 163000, Russia; andrej.grjibovski@gmail.com (A.M.G.); vipostoev@yandex.ru (V.A.P.); 3 Department of Biochemistry and Biomedical Sciences, McMaster University, Hamilton, ON, L8S 4L4, Canada; nieboere@mcmaster.ca; 4 Department of Preventive Medicine, International Kazakh -Turkish University, Turkestan 050040, Kazakhstan

**Keywords:** screening, cervical cancer, uterine, survival, Arkhangelsk Cancer Registry, Russia

## Abstract

The aim of the current study was to compare cervical cancer (СС) patients diagnosed with and without screening in terms of: (i) sociodemographic and clinical characteristics; (ii) factors associated with survival; and (iii), and levels of risk. A registry-based study was conducted using data from the Arkhangelsk Cancer Registry. It included women with newly diagnosed malignant neoplasm of the uterine cervix during the period of 1 January 2005 to 11 November 2016 (N = 1548). The Kaplan-Meier method, the log-rank test, and Cox regression were applied. Most participants who were diagnosed by screening were at stage I and died less frequently from CC than those diagnosed without screening. The latter group was also diagnosed with СС at a younger age and died younger. Younger individuals and urban residents diagnosed with stage I and II, squamous cell carcinoma had longer survival times. Cox regression modeling indicated that the hazard ratio for death among women with CC diagnosed without screening was 1.61 (unadjusted) and 1.37 (adjusted). CC diagnosed by screening, cancer stage, patient residence, histological tumor type, and age at diagnosis were independent prognostic variables of longer survival time with CC. Diagnosis of CC made within a screening program improved survival.

## 1. Introduction

Cervical cancer (CC) ranks as the second-to-third most frequent cancer in women worldwide with an estimate of 528,000 cases in 2015 [1,2]. In 2015, about 266,000 women died from this preventable disease [1]. The incidence and death rates are substantially higher in low- and middle-income countries due to limited access to preventive measures [2]. In Russia, CC ranks as the fifth leading cause of female cancer with an estimate of about 15,342 new cases diagnosed annually [3]. Its crude incidence rate in Russia is generally lower than in Arkhangelsk region (21.27 versus 24.25 in 2015) [4]. The Arkhangelsk region is the biggest Arctic region in Europe and its Gross Regional Product (GRP) places it as a middle-income region in the Russian Federation [5].

The objectives of CC screening are to detect precancerous lesions and early stage cancer, thereby avoiding new cancer cases and the development of advanced stages and deaths from them [2]. Over the last 50 years, the incidence and mortality rates of CC have shown remarkable reductions in countries with organized cytology-based screening programs [6,7]. The introduction of screening appears to have improved survival [8]. Opportunistic screening (i.e., screening on demand) can also decrease CC rates, but usually to a smaller extent [9,10]. This approach tends to cover younger women, thereby often missing those at highest risk [11], and success depends strongly on attendance rate. Generally speaking, non-participation in screening is associated with a lower level of education [11,12,13], single status [12,14], smoking [12], and thus a low awareness level is likely [15,16]. Ethnicity [17], psychological barriers [18], and residence (urban/rural residence) are other factors that have been shown to be associated with participation rates in screening programs [19].

Canada and the United States have been regarded as leaders in cervical cancer screening. Most of the research on the implementation of screening has taken place in US settings, where it is predominantly opportunistic. In Canada, a combination of opportunistic and organized approaches is used [20,21]. The latest recommendations by the European Union (EU) state that cancer screening programs should only be offered on a population basis in an organized fashion, and must include quality control protocols at all levels [22]. National and World Health Organization (WHO) guidelines that describe how to start and organize a screening program are available [23]. There is wide variation in the structure of СС screening programs, and this reflects the resources available [19]. Cancer-screening programs in the Nordic countries have been successful in decreasing incidence and mortality rates. Finland was the first country to have success in organized screening of СС. Its implementation has resulted in rapid decreases in the invasive СС incidence and mortality rates. It also has generated a change in the staging and histological distribution of СС in that country [24].

In spite of existing screening methods, СС continues to be a major public health problem in the Russian Federation [25]. The incidence and mortality rates of СС in Russia are generally high and show little tendency to decrease [25]. Cytological screening for this cancer was introduced in the Soviet Union in 1964 [26]. Until 2003 the screening was opportunistic, and was based on conventional cytology stained by the Romanovski-Gimse method with Ayre’s spatula as the tissue sample-taking instrument. In 2003, the Ministry of Health of the Russian Federation issued order No. 50, which delineates the preferred CC screening procedure [27]. The order states that cytological testing for CC should be started at the age of 18 with no upper age limit, is to be performed annually, and should be combined with a gynecological examination. The cytobrush is the preferred instrument for acquiring samples, when possible. In 2012 the Ministry of Health of the Russian Federation issued a new order (No. 572n), which included standards for medical care in the field of obstetrics and gynecology [28]. This order did not specify instructions on screening, its frequency, a recommended age of initiation, nor specific tissue sampling instruments and staining methods. Its focus was on the International Classification of Disease (ICD) cancer codes. There is no national screening registry in Russia, nor guidelines for the management of women with cervical pathology. Currently, Human papilloma virus (HPV) testing and vaccination are available on demand for a fee, but are not included in the national vaccine calendar. The Romanovski-Gimse staining method is still widely used in the region. It was in this context that a regional cancer registry was set up in Arkhangelsk, Northwest Russia.

Information on survival is recognized as an important indicator in cancer control activities [29]. Survival analyses provide important information for public health authorities in evaluating treatment access and its effectiveness. We employed data from the Arkhangelsk Cancer Registry (ACR) to compare CC patients diagnosed with and without screening in terms of: (i) sociodemographic and clinical characteristics; (ii) factors associated with survival; and (iii) calculated hazard ratios.

## 2. Materials and Methods

### 2.1. Study Setting, Design, and Sample Size

The Arkhangelsk Region (AR) is located in the northwestern part of the Russian Federation. It covers an area of 589,900 square km and had a population of 1,174,078 on 1 January 2016 [5]. The ACR is a joint effort of the University of Tromsø (Norway) and the Arkhangelsk Regional Oncological Hospital, and was established in 1999. Our data include all cases of cervical cancer registered during the period of 1 January 2005 to 11 November 2016. Electronic registration of cancer cases within the ACR consists of three databases. The ACR contains the following information: date of birth, sex, ethnicity, maternal occupation, date of diagnosis, ICD-9 and -10 codes, histological tumor type, morphology code, stage, tumor, node and metastasis (TNM) stage, method of cancer diagnosis, how the tumor was revealed, type of treatment and its result, the occurrence of cancer metastases, and cancer recurrence. Additional details about its content and implementation have been described previously [30]. Data quality control exercises of the ACR were conducted twice, specifically in November 2003 and May 2003, and the quality of the registered information was accepted as suitable for epidemiological studies [30]. According to the Russian legislation (order No. 135, issued by the Russian Ministry of Health on 19 April 1999), every newly diagnosed cancer has to be reported by physicians to an oncological hospital within three days using a prescribed form. Three trained individuals entered the received data into the Cancer Registry database. Since 2000, a computer program was installed to monitor the accuracy of the entered data [30].

A total of 1940 CC cases were registered in the ACR for the designated period. The inclusion criterion was the presence of a newly diagnosed malignant neoplasm of the uterine cervix. The study sample included 1548 cases, with 371 being excluded due to repeated consultations (cancer recurrences), and 21 were lost to follow-up.

### 2.2. Data Collection

Age at the time of diagnosis and of death were presented as continuous variables (in years). For the purpose of the survival analyses, we used the International Cancer Survival Standard (ICSS) weights for CC, with age at diagnosis divided into five groups: 15–44 years, 45–54 years, 55–64 years, 65–74 years, and over 75 years [31]. Vital status by the end of each year was categorized as: (i) death from CC; (ii) death from other reasons; and (iii) alive. Survival time was calculated in months, with the day of diagnosis as the initial date. For patients whose underlying cause of death was CC, the final date was that at death. Deaths from other causes were also recorded in the ACR, including its date of death. For patients who did not die, the final date of the study period was 11 November 2016. Stage-specific survival analysis was carried out for each stage separately. Furthermore, data for stages I and II were combined to yield an early cancer variable, and that for stages III and IV were combined for advanced cancers. This was done because of the small number of observations.

Based on medical records, the ACR contained information on CC diagnostic methods (whether with or without screening), cancer location and stage, year-end vital status, histological type of tumor, age at diagnosis, date of birth, and date of death (if applicable). Residence was defined as urban or rural. Diagnosed with screening means that the diagnosis was made during a regular health check or when consulting a gynecologist for a condition not related to symptomatic patient care. Regular health checks are usually done over the non-selected population, i.e., people belonging to different age groups, residences, and occupations. They routinely include testing for cervical cancer (cytological sampling). A diagnosis made without screening indicates that the patient was symptomatic for CC. The most common symptoms are copious foul-smelling vaginal discharge, abnormal bleeding or inter-menstrual bleeding, postcoital bleeding, postmenopausal bleeding, or backache. In both settings the cervical cytology was performed similarly in the diagnostic process. Cancer stage at diagnosis was in accordance with the guidelines of the International Federation of Gynecology and Obstetrics (FIGO). Patients with an in situ cancer were not included in the registry, nor in the survival analyses. Histologic subtypes were classified according to the International Classification of Disease for Oncology, 2nd ed. (ICD-02). For purpose of analysis, histopathological types were grouped as squamous cell carcinomas (codes 8050–8078, 8083–8084); adenocarcinomas (8140–8141, 8190–8211, 8230–8231, 8260–8263, 8310, 8380, 8382–8384, 8440–8490, 8570–8574, 8576), and other/unspecified malignant neoplasms (8010–8380, 8382–8576, 8010–8035, 8800–8811,8830, 8840–8921, 8990–8991, 9040–9044, 9120–9133, 9150, 9540–9581, 8000–8005).

### 2.3. Data Analysis

We used the Pearson’s chi-squared test to analyze categorical variables. The *t*-test was used to compare continuous variables, the Kaplan-Meier method approach was applied in the determination of CC mean survival times and related graphics, the log-rank method was used for the comparison of accumulated survival curves, and the Cox proportional risk model was employed for the calculation of hazard ratios (HRs) for the independent study variables. The latter were adjusted for age, cancer stage and histology, residence, and year of diagnosis. The statistical analyses were carried out using SPSS version 24 (SPSS Inc., Chicago, IL, USA).

### 2.4. Ethical Considerations

This study was granted ethical approval by the Ethical Committee of Northern State Medical University, Arkhangelsk, Russia (Этический комитет Северного Государственного Медицинского Университета), Report Number 01/02/2017 obtained on 01/03/2017; and by the Norwegian Regional Committee for Medical and Health Research Ethics (RECNorth), Tromsø, Norway, Registered Report Number 2014/1670.

This study was based on a retrospective review of the Arkhangelsk Cancer Registry (ACR) de-identified records. Informed consent for this study was not obtained because our study database does not contain personal information and the project was not interventional. The cancer reporting is specified in the Russian legislation. Related specifics have been described by Vaktskjold et al. [30].

## 3. Results

There were 1940 cases of confirmed and registered primary invasive cancers of the cervix. Among these, 1548 records matched the selection criteria and represent the study sample. Most of the cases were diagnosed at stage I, squamous cell carcinoma was the predominant histological form, and one third of deaths were due to CC (see Table 1). Residence, age at diagnosis, cancer stage, tumor histology, and the number of deaths among those diagnosed with the screening are also reported in this table. Most participants who were diagnosed by screening were at stage I (*p* < 0.001), and died less frequently from CC (*p* < 0.001) than those diagnosed without screening. The latter group was also diagnosed with CC at a younger age (*p* = 0.013) and died younger (*p* = 0.002). Compared to women with CC diagnosed by screening, tumor histology and residence did not differ for those diagnosed without screening (Table 1).

**Figure 1:**
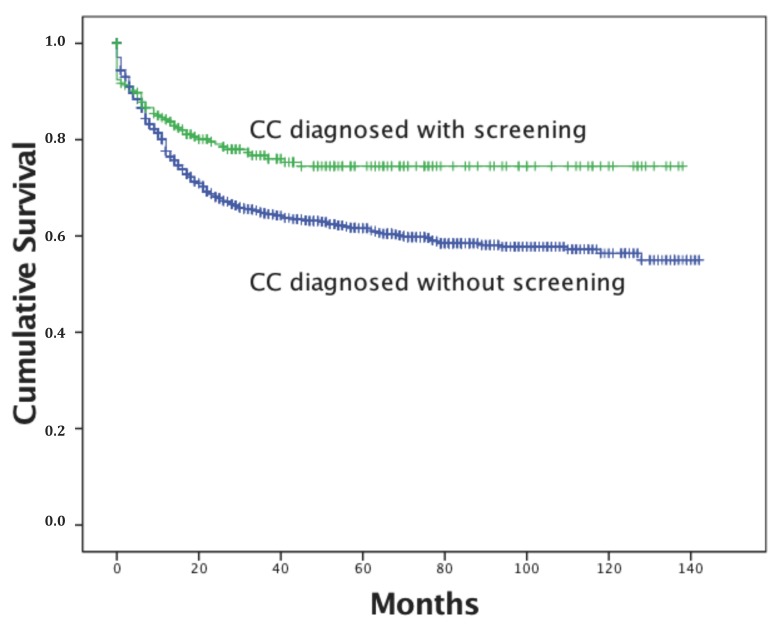
Survival curve for women with cervical cancer and registered in the Arkhangelsk Cancer Registry (ACR).

**Figure 2:**
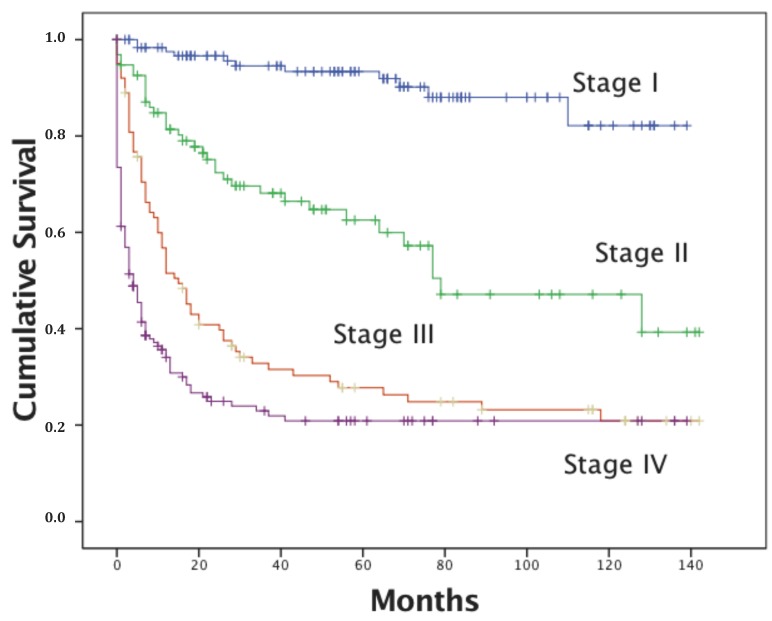
Survival curve for women with different stages of cervical cancer registered in the ACR.

The Kaplan-Meier survival curves for the study period are provided in Figure 1 and illustrate a significant difference in survival time between the two groups (*p* = 0.001). The 5- and 10-year survival was about 60% among CC patients diagnosed without screening and more than 70% for those diagnosed with it. In Figure 2, the Kaplan-Meier curves are depicted by initial staging. Five-year survival was about 97% for stage I, 64% for stage II, 38% for stage III, and 19% for stage IV.

In stage-specific analyses, we observed significant difference in survival for those diagnosed with screening compared to those diagnosed without only for stage II (*p* = 0.052); while for stage I *p* = 0.379, for stage III *p* = 0.495, and for stage IV *p* = 0.789.

**Figure 3:**
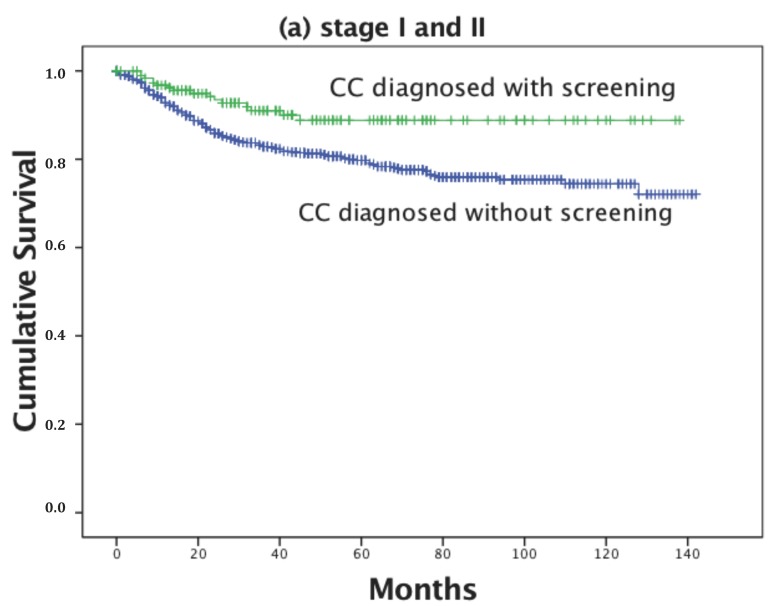

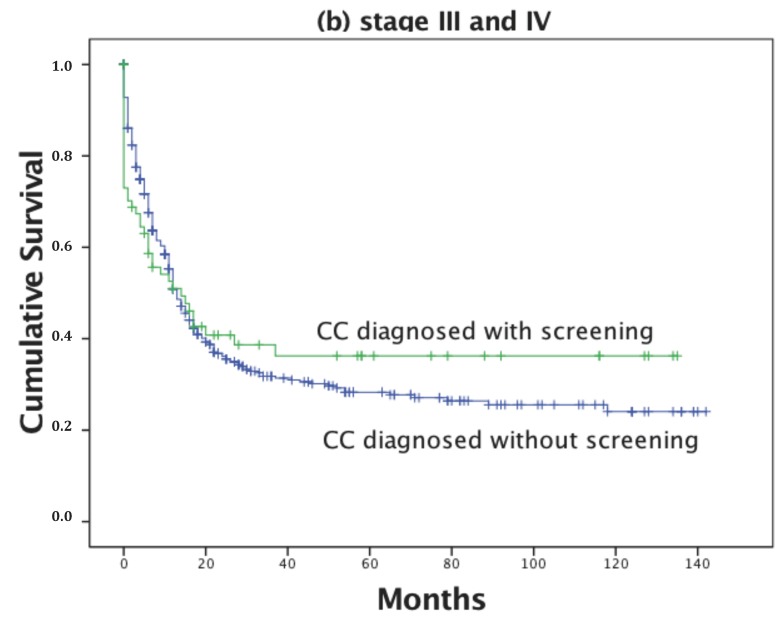
Stage-specific survival curves for women diagnosed with different types of CC (whether with or without screening) and registered in the ACR: (**a**) stages I and II (*p* = 0.003); (**b**) stages II and III (*p* = 0.890).

As illustrated in Figure 3a,b, women diagnosed with CC with screening in the early stages (I and II) of this disease survived longer when compared to those diagnosed without it. However, for the advanced stages (III and IV), we did not find such a difference.

The sociodemographic and clinical variables for the unadjusted survival functions are provided in Table 2.

At the end of the follow-up, 59 (22.5%) and 455 (35.4%) of women, respectively with CC diagnosed with and without screening, had died. Younger aged, urban residents diagnosed with stage I and II, squamous cell carcinoma had somewhat longer survival times. Cox regression modeling indicated that the hazard ratio for death among women with CC diagnosed without screening was 1.61 (unadjusted) and 1.37 (adjusted) (Table 3).

## 4. Discussion

### 4.1. Main Findings

Our data show that death from CC among women with diagnoses made without opportunistic screening after adjustment for cancer stage, patient residence, histological tumor type, age, and year of diagnosis was 37% higher compared to those diagnosed with screening. Women diagnosed with CC by screening in the early stages (I and II) survived longer when compared to those diagnosed without screening. However, for the advanced stages (III and IV), we did not find such a difference.

### 4.2. Data Interpretation and Comparisons with Previous Studies

The observed mean age at diagnosis of СС was 48.5 years, which is comparable to values reported by others. For example, in a British study [32] the median age at diagnosis for CC approached 50 years. Screening ages between the late 40s to middle 60s appear to be most common. Our findings of a significant difference in the mean age of CC cases diagnosed with and without screening (48.1 and 50.6 years, respectively) is consistent with that observed in a Swedish study [33]. The latter showed a slight increase in the mean age at diagnosis for all stages after the introduction of screening. By contrast, others report no significant differences in the median age at diagnosis [32,34,35]. The age of screening initiation varies from country to country. In the Russian Federation, national screening protocols are regulated by the Ministry of Health Orders No. 50 and 808, which specify that CC screening should be started at the age of 18, or at initiation of sexual activity with no upper age limit. Initiation of screening at an early age can lead to overestimation of CC risk. Landy et al. concluded that screening from age 20 years on would lead to over-treatment and over-testing, while having little impact on CC prevention [36].

Our data show that residence was not associated with CC diagnosis made with or without screening. By contrast, lower participation rates in CC screening have been reported for rural areas of the USA [37]. Rural residents would appear to have a higher risk of late cancer detection due to barriers that include: lack of convenient access to or availability of preventive health services (including early detection screening) [38,39,40] and a lack of awareness and knowledge about the existence of screening programs [41,42]. On the other hand, for cancers diagnosed at late stages, the absence of significant associations between rural/urban residence and survival [43] have been reported [44].

The fraction of the participants with a positive CC diagnosis decreased across the four CC stages, namely: 39.1% (I), 26.1% (II), 22.7% (III), and 12.0% (IV). At stage I, the percent of CC cases diagnosed with screening was higher compared to those without (51.3 versus 36.7%). This is consistent with the findings of Hellman et al. [33], who reported an increase in stage I to more than 50% of all CC cases. Several studies presented opposite results. For example, Nowakowski et al. [34] reported that advanced stages of CC dominated in a cervical screening program in Poland. In the present study, women diagnosed with CC with screening (stages I and II) had longer survival compared to those diagnosed without screening. However, for the advanced stages we did not find this difference. This may be partly explained by the speedier examination of those diagnosed with screening. Usually, women diagnosed without screening have to wait for a colposcopy and biopsy appointment, which could take up to six months.

After the collapse of the Soviet Union, the number of primary healthcare centers and number of medical workers has decreased in Russia, and in about 17,500 municipalities there is no health infrastructure. Moreover, 35% of settlements are not covered by public transport and ambulance services are often not available [45]. The latter may be one possible explanation for the low level of participation in CC screening programs. Several factors determine the participation in preventive measures for CC: (i) at the system level, there is underfunding; (ii) at the provider level, suitable screening intervals are not recommended, nor is treatment/follow-up carried out in a timely manner; and (iii) at the individual level, women often face lack of transportation/childcare, impeding clinical visits, and have insufficient knowledge about screening. A 2010 Norwegian study illustrated the importance of such knowledge, including awareness of screening intervals and CC risk factors for enhancing public participation [14]. Sporadic screening or lack of communication among healthcare professionals that lead to misunderstanding between cytologists and gynecologists might have been responsible, as well as low screening coverage (on average 43–45%, with a range of 11.5–61.9% in 2009 and 23.6–24.6% in 2001–2007). Furthermore, low attendance rates across a region due to the low awareness of women about the risk of CC might contribute as well [46,47]. Lack of training in smear sampling and usage of old instruments have also been identified as possible reasons for screening failure in a region [48], as well as demographic changes in population size and distribution by age and sex [25]. It has been reported that one fifth of the patients diagnosed with CC in the Republic of Karelia died within the first year of the disease [46].

Squamous cell carcinoma was the predominant histological type and accounted for more than 80% of all CC cases, of which 9.1% were adenocarcinoma. During the 1950s and 1960s worldwide, nearly 95% of all invasive CCs were squamous cell carcinomas, followed by adenocarcinomas (5%) [49,50]. Most recently, the squamous type accounted for approximately 75% of CC, whereas adenocarcinomas contributed 25% [51]. This change is likely due to the introduction of screening [32] with cytological testing as the primary screening tool [49,52]. The difficulty of anatomic accessibility has been suggested as one of the main causes for the low detection rates and the diagnosis of late stage adenocarcinomas [53]. Therefore, one of the methods suggested for improving early detection of adenocarcinoma is to use a combination of cytology and testing for high-risk HPV types [52]. The healthcare system availability, wide use of contraceptive pills, and changes in smoking habits and in sexual behavior increased awareness of CC risk all appear to have contributed to the changes in CC distribution by age, stage, and histopathology [33].

Our study group of women diagnosed with CC with screening had higher 5-year survival times than those who were diagnosed without screening. Worldwide, the 5-year survival from CC varies widely from <50% to >70%, although in most countries it has increased somewhat during the last 10 years. The highest 5-year survival reported occurred in the Nordic countries (78%), while the lowest was registered in Malta (44%) [54]. According to the American Cancer Society (ACS) in 2010, the overall 5-year survival in the USA was 72% [55]. While the 5-year survival for our stage I patients concurs with that reported by the ACS (≤93%), for the other three stages they were lower than their values of ≤63% (stage II), ≤35% (stage III), and around 15% (stage IV) [55].

Age as a prognostic factor for survival can be confounded by age-dependent factors, since the prevalence of other diseases (e.g., hypertension and cardiovascular diseases) may mitigate receiving optimal treatment or a favorable result from it [56]. Survival up to 87% for women aged less than 30 years and 45.5% for those older than 70 years is typical [56]. In the EUROCARE-3 study, the relative survival in the age group 15–44 years at diagnosis was more than two-fold higher when compared to the group of women aged 75 or more (74% and 34%, respectively at 5 years). Our findings closely match the latter (Table 2).

According to the International Agency for Research on Cancer (IARC) [35], the CC stage at diagnosis is generally the most important factor in the survival of cancer patients [56,57]. In our study, women with late-stage CC had substantially lower survival rates, even less than 5 years after first CC diagnosis (Figure 2). Improvement in survival is often used as an indicator of successful screening. In Finland, the implementation of screening for cervical cancer resulted in a slight decrease in survival [58]. The latter was attributed to a growing proportion of cases of advanced cancers in women not attending screening. However, most counties in the study that showed improvements in survival of CC had adequate diagnostic and treatment procedures and screening programs in place.

### 4.3. Limitations and Strengths

To the best of the authors’ knowledge, this study is first in Russia to use the cancer registry data to assess the effectiveness of CC screening and patient survival. Our analysis is limited by the lack of data on socioeconomic status, smoking, use of oral contraception, parity, and cases of carcinoma or adenocarcinoma in situ—the precursors of invasive CC. The results of the cytological screening were not available and could not be included in the analysis. Information about HPV type-specific infection that would have helped to quantify its role in the development of specific histologic CC subtypes was also not available. Therefore, our study might have been biased by over-diagnosis. The appearance of the latter is common in early detection programs [59]. Another related and common phenomenon in screening programs is healthy-volunteer bias, because individuals willing to participate in screening may be of a higher socioeconomic group, have easier access to healthcare, and follow the health providers’ recommendations more precisely. Consequently, those who participated in screening may have lower mortality rates and longer survival than the total population at risk [56]. Lead-time bias may also have been an issue. Early diagnosis by screening may not improve survival and could enhance a patient’s anxiety as she needs to live longer with the knowledge of having CC. To avoid this, more sensitive screening tools have to be designed and used to ensure that the diagnosis can be made at the pre-cancer stage.

## 5. Conclusions

Our study demonstrates that death from CC was 37% higher when the diagnosis was made without opportunistic screening when compared to those diagnosed with screening after adjustment for cancer stage, patient residence, histological tumor type, age at diagnosis, and by year. The hazard rates for death among women with CC diagnosed without screening were also significantly higher compared to those diagnosed with it. Diagnosis of CC made within our screening program further prolonged survival. Exploring women’s awareness about existing CC screening programs should be considered in efforts to enhance participation rates. In terms of generalizability, our results may well apply to other regions of Russia because of the similarity of CC screening programs to that described.

## Figures and Tables

**Table 1:** Sociodemographic characteristics and clinical variables of women with different types of cervical cancer diagnostics.

Variables	N (%)	No Screening	Screening	*Р ^1^*
		N = 1285	N = 263	
*Age at Diagnosis (years) ^2^: Mean (SD)*	48.5 (15.2)	48.1 (15.1)	50.6 (15.7)	0.013
*Residence:*				0.145
urban	1122 (72.5)	941 (73.2)	181 (68.8)	
rural	426 (27.5)	344 (26.8)	82 (31.2)	
*Stages:*				<0.001
I	606 (39.1)	471 (36.7)	135 (51.3)	
II	404 (26.1)	349 (27.2)	55 (20.9)	
III	352 (22.7)	314 (24.4)	38 (14.4)	
IV	186 (12.0)	151 (11.7)	35 (13.4)	
*Histological tumor type:*				0.829
squamous cell	1292 (83.5)	1072 (83.4)	220 (83.7)	
carcinoma	141 (9.1)	116 (9.0)	15 (9.5)	
adenocarcinoma unspecified/other	94 (6.1)	78 (6.1)	16 (6.1)	
uknown	21 (1.4)	19 (1.5)	2 (0.7)	
*Vital status:*				<0.001
alive	969 (62.6)	773 (60.2)	196 (74.5)	
died from cervical cancer	514 (33.2)	455 (35.4)	59 (22.4)	
died from other causes	65 (4.2)	57 (4.4)	8 (3.0)	
*Age of death (years) ^2^: Mean (SD)*	54.2 (16.5)	53.5 (16.3)	60.3 (17.3)	0.002

^1^ Chi-square test; ^2^ *t*-test.

**Table 2:** Number of deaths, mean survival time and its 95% confidence lower limits (LL) and upper limits (UL) estimated by the Kaplan-Meier method, and the results of the log-rank test (*p*-value) for the study variables in women with cervical cancer.

	N	Deaths	Mean Survival Time (Months)			Log-Rank *p*-Value
			Mean	95% LL	95% UL	
*Cervical cancer (CC) diagnosed by screening:*						0.001
no	1285	455	89.7	85.9	93.4	
yes	263	59	105.8	98.5	113.0	
*Age in years:*						<0.001
15–44	746	202	101.7	97.0	106.4	
44–54	314	99	94.1	86.5	101.7	
55–64	244	93	80.6	71.2	90.0	
65–74	158	60	82.8	72.2	93.1	
75 and more	107	60	53.4	40.7	66.0	
*Stage:*						<0.001
I	606	36	132.5	129.5	135.5	
II	404	135	92.2	85.6	98.9	
III	352	203	55.7	48.5	62.9	
IV	186	140	30.7	22.4	39.0	
*Residence:*						0.018
urban	1122	358	95.0	91.0	98.9	
rural	426	156	85.9	78.9	92.8	
*Histological tumor type:*						<0.001
squamous cell carcinoma	1292	390	97.1	93.4	100.7	
adenocarcinoma	141	66	67.7	55.2	80.2	
unspecified/other	94	42	73.6	59.8	87.5	
unknown	21	16	31.6	11.3	51.8	

**Table 3:** Hazard ratio (Cox model, crude and adjusted), with the respective lower and upper 95% confidence limits (LL and UL) in women with cervical cancer.

	Crude			Adjusted ^1^		
	HR	95% CI	*p*-Level	HR	95% CI	*p*-Level
*Cervical cancer diagnosed by screening:*						
no	1.61	1.22–2.10	0.001	1.37	1.04–1.80	0.027
yes	1.00			1.00		

^1^ Adjusted for age, cancer stage and histology, residence, and year of diagnosis.
